# Post-discharge nutritional management for patients with coronary heart disease and frailty: a qualitative study

**DOI:** 10.1186/s12877-024-04885-7

**Published:** 2024-03-19

**Authors:** Yifei Yang, Jing Gong, Binxu Yang, Chan Chen, Xintong Deng, Kejun Chen, Yingying Zhao, Xusihong Cai, Jingjing Li, Jing Zhou

**Affiliations:** 1 The Second Affiliated Hospital of Zunyi Medical University, Zunyi, Guizhou, China; 2 School of Nursing, Zunyi Medical University, Zunyi, Guizhou, China

**Keywords:** Coronary heart disease, Frailty, Nutrition management, Qualitative study

## Abstract

**Background:**

Frail elderly patients experience physiological function and reserve depletion, leading to imbalances in their internal environment, which increases the risk of coronary heart disease recurrence and malnutrition. However, the majority of these patients, who primarily have a low level of education and lack self-management skills, face difficulties actively dealing with obstacles during the transition period after their discharge from hospitalization. Therefore, it is necessary to understand and discuss in depth the nutrition management experience of discharged elderly patients with coronary heart disease and frailty (ages 65-80 years old) and to analyze the promoting and hindering factors that affect scientific diet behavior during the discharge transition period.

**Methods:**

Fifteen elderly patients with coronary heart disease and frailty who had been discharged from the hospital for 6 months were interviewed using a semistructured method. The directed content analysis approach to descriptive research was used to extract topics from the interview content.

**Results:**

All participants discussed the problems in health nutrition management experience of discharged. Five topics and ten subtopics were extracted, such as ①Weak perceptions and behaviors towards healthy eating (personal habit solidification, negative attitudes towards nutrition management), ②Lack of objective factors for independently adjusting dietary conditions (reliance on subjective feelings, times of appetite change), ③Personal hindrance factors (memory impairment, deficiencies in self-nutrition management), ④Expected external support (assistance care support, ways to obtain nutritional information), ⑤Lack of continuous nutrition management (interruption of professional guidance, avoidance of medical treatment behavior).

**Conclusions:**

Nutrition management after discharge places a burden on elderly patients with coronary heart disease and frailty. According to the patients' physical conditions, we should develop a diet support system that is coordinated by individuals, families and society.

## Background

Frailty is a multidimensional syndrome that occurs specifically in the elderly and is usually characterized by a cumulative decrease in the patient's physiological functional reserve, leading to a significant limitation of physical activity on the part of the elderly individual [1]. In elderly patients with coronary heart disease, this restricted activity leads to loss of muscle mass and abnormalities in body metabolism, features that are closely associated with the development of frailty in old age [2]. Studies have shown that the incidence of frailty in elderly patients with coronary artery disease ranges from 18.8% to 57.8%, which can increase their readmission rates by a factor of 4.3 [3] and the incidence of malnutrition among such patients by a factor of 6.841 [4]. Leading to challenges in the quality of life of discharged elderly patients with coronary heart disease and frailty. Studies have shown that nutritional deficiencies in older adults lead to reduced muscle mass, decreased grip strength, increased fluid accumulation, and depleted amino acid and energy levels, which in the long term can lead to reductions in cardiac muscle mass and skeletal muscle mass [5]. Therefore, the treatment of coronary heart disease and frailty should be considered jointly to improve the quality of life of patients.

In 2022, the Office of the Chinese Working Committee on Aging issued a notice on the implementation of nutrition improvement actions for elderly individuals, with the aim of improving the nutritional health literacy of elderly individuals and improve their quality of life by strengthening nutritional interventions for the elderly [6]. Although an increasing number of health care professionals are beginning to pay attention to the nutritional status of elderly patients with coronary heart disease and frailty, the low-salt and low-fat diet structure of elderly patients with coronary heart disease who had been discharged from the hospital for 1 year was evaluated, and patients who could not adhere to the low-salt and low-fat diet structure accounted for 68.5% of the total, indicating that the nutritional compliance of elderly patients after discharge from the hospital is not satisfactory [7].It is evident that in order to enhance nutrition management adherence for patients with coronary artery disease complicated by frailty post-discharge, it is imperative to first understand how they navigate the transition period after discharge, adapting to changes in their roles and environment. Identifying the factors that influence the effectiveness of nutrition management during this transitional period is essential.

Meleis’ transition theory aims to provide researchers with a clearer understanding of the complex transition process of study subjects, identify patient needs and potential risks, and develop interventions to facilitate smooth transitions; this theory mainly includes the nature of the transition, the conditions of the transition, response patterns, and nursing intervention [8]. Meleis’ transition theory has mainly been applied to nursing education transitions [9], caregiver role transitions [10], nurse position changes [11] and other fields. Elliott M [12] et al. conducted qualitative interviews with parents of children with precardiac disease and used Meleis’ transition theory framework to guide the formulation of interview questions and analysis of the results with the goal of obtaining insights into caregivers' experiences of transition and providing theoretical support for interventions that can be implemented by patients themselves, caregivers, and society. Eyimaya [10] et al. explored the characteristics of transition among menopausal women and used Meleis’ transition theory as a framework. These authors explored in depth the nature of the transition, the conditions of the transition, response patterns, and corresponding nursing interventions associated with this population as a way to reduce the severity and negative impacts of menopausal symptoms in women.

Most previous studies have focused on the use of questionnaires to investigate the knowledge, abilities and attitudes of caregivers in the context of nutritional care [13, 14] and have not analyzed the specific experiences of elderly patients themselves in the process of dietary management. Therefore, we aim to utilize Meleis’ Transition Theory to delve into the post-discharge nutrition management needs of coronary heart disease patients and frailty. We structured the interview guide using the nature of the transition, the conditions of the transition, response patterns mechanisms as a framework, covering various aspects including experiences, changes and differences, individual factors, community factors, social factors, process indicators, outcome indicators, and more. By uncovering the post-discharge nutrition management experiences of these patients, we hope to organize these findings into specific nursing intervention strategies. This process completes the cycle of Meleis’ Transition Theory, laying the foundation for future nutritional interventions.

## Materials and methods

### Study design and theory framework

The aim of our research is to explore the experiences of nutrition management in patients with coronary heart disease and frailty after discharge, based on Meleis Transition Theory. In this study, patients with a combination of coronary heart disease and frailty who had been discharged from the hospital for more than 6 months were recruited as interviewees, and their personal, family, and external support as well as hindrances to their nutrition management were explored by collecting their experiences of nutrition management during the process of discharge from the hospital. To make the interviews more structured and increase their depth, this researched employed a theory-based, qualitative design and used the theoretical framework of Meleis’ transition theory to develop an outline for a semistructured interview (Table 1).

**Table 1:** Design of interview outline

Aspect of Meleis' Transition Theory	Code	Interview Question
Nature of the transition	Experience	Do you feel differently when you manage your diet in the hospital and at home, and is there a difference in dietary intake? In what specific ways? What are the reasons?
	Key events	How did it feel to adhere to a managed diet after being discharged from the hospital? What periods were the most stressful?
Conditions of the transition	Facilitation and inhibition	What bothered you the most about eating after you were discharged from the hospital?
	Family support	Do you manage your diet together with your family members, and if so, how is the work divided?
Response patterns	Coping style	How do you solve the problem of disruptions to your regular diet?
	Desired support	Have you learned about proper diet before? How has it helped you? If not, what knowledge would you most like to receive?

Prior to the formal interviews, preliminary interviews were conducted with three discharged patients. Based on their responses, it was found that the interview outline was not able to fully extract their feelings about nutritional management after discharge in terms of external support and outcome control. Additionally, the questions prepared in the interview outline did not specifically address the dimensions of Meleis Transition Theory. Therefore, the interview outline was adjusted (Table 2) to finalize the main points for the interviews.

**Table 2:** Formal interview outline

Aspect of Meleis' Transition Theory	Code	Interview Question
Nature of the transition	Experience	Do you feel differently when you manage your diet in the hospital and at home, and is there a difference in dietary intake? In what specific ways? What are the reasons?
	Variation and Difference	How did it feel to adhere to a managed diet after being discharged from the hospital? What periods were the most stressful?
Conditions of the transition	Personal Factors	What bothered you the most about eating after you were discharged from the hospital?
	Community Factors, Social Factors	Do you and your family members manage your diet together? If yes, how is the work divided? If not, what kind of nutritional management support would you like to receive from the outside?
Response patterns	Process Indicators	How do you solve the problem of disruptions to your regular diet?
	Outcome Indicators	What feedback do you think gives you better control over your nutritional management?

### Setting and sample/participants

Fifteen elderly patients who had been discharged from the Second Affiliated Hospital of Zunyi Medical University with a combination of coronary heart disease and frailty were interviewed.

#### Inclusion criteria

 ① aged 65–80 years; ②The coronary angiography diagnoses the degree of coronary atherosclerosis as 50% to 70%; ③ FRAIL frailty phenotype score is ≥ 1; ④ discharge time before May 2022; ⑤ provision of informed consent and voluntary participation in this study.

#### Exclusion criteria

 ① diagnosis including hepatic and renal insufficiency, malignancy, thyroid disease, peptic ulcer, immune system disease, or hyperuricemia; ② inability to communicate normally.

#### Informed consent

 The purpose of the interview was explained to the patients prior to the interviews, and consent was obtained from the patients.

### Data collection

Both researchers were trained in a rigorous qualitative research course prior to the interviews to ensure that the researchers had good interviewing skills and that the interviews were conducted smoothly. One researcher served as the main interviewer, and another researcher performed the recording and transcription work. The interviewees were contacted by telephone to explain the purpose of the interview and to obtain their consent before the interviews, which were conducted by telephone or video; each interview lasted approximately 20–40 min.

Researchers inquired about the dietary conditions of the interviewees and their family members one week prior to the interview, conducting evaluations and education on the interviewees’ balanced diet, consumption diversity, high-quality protein intake, and fat intake. Based on an understanding of the interviewees’ individual dietary habits, targeted dietary management guidelines were proposed. During the communication process, the FRAIL scale was used to inquire about the patients’ frailty status, including: ① Feeling fatigued in the past four weeks; ② Difficulty climbing a flight of stairs without the aid of tools or others’ assistance; ③ Difficulty walking 500 m without the aid of tools or others’ assistance; ④ Having more than five diseases; ⑤ Losing more than 3 kg in weight in the past six months (not due to dieting or exercise). After communicating with the patients to understand their basic situation, establishing trust, conveying the purpose of the interview, and ascertaining the patients’ willingness, the formal interview on their nutritional management experiences post-discharge commenced.

### Data analysis

The researchers combined the collected audio recordings and written descriptions to create a written version of the interview records, which were then imported into NVivo software. Using a directed content analysis approach [15], the researchers analyzed the interview data according to the three components of Meleis’ transition theory: the nature of the transition, the conditions of the transition, response patterns. The specific steps included: extracting categories such as participation, change, individual factors, community factors, social factors, establishing connections, interaction, orientation, sense of control, and sense of belonging based on Meleis’ transition theory and explaining the significance of each category; reading the data word by word to highlight content similar to various concepts of Meleis’ transition theory and categorizing them; assigning new codes to unclassifiable data and adjusting existing coding schemes; categorizing codes into sub-themes based on their relevance and relationships; organizing and conceptualizing sub-themes into themes; and presenting the results to the participants for verification to enhance the validity of the study. During the data analysis process, to determine the dependability of the classification, two researchers explained and analyzed each other's views. When these researchers expressed different views, a third expert was invited to discuss them.

## Results

### Participant profile

Table 3 shows the demographic characteristics of the 15 discharged elderly patients with coronary heart disease and frailty who were interviewed, including 6 males and 9 females; participants’ mean age was 71 years (SD 4.4). In terms of frailty scores, the participants were predominantly prefrailty (*n* = 10). Among all older patients, 10 lived in cities, and 5 lived in rural areas. With regard to their level of education, 8 elderly participants had a primary school education or below, Regarding length of discharge, 4 were discharged for 6 and 8 months, respectively, and the rest have only one.

**Table 3:** Participants’ general information

Identification number	Gender	Age	Place of residence	Length of hospital stay (months)	Frailty score
P1	Female	75	Rural	9	3
P2	Female	69	Rural	10	2
P3	Female	78	Urban	8	3
P4	Female	73	Urban	11	2
P5	Female	78	Urban	8	3
P6	Male	69	Rural	7	2
P7	Female	76	Rural	6	3
P8	Male	74	Urban	6	1
P9	Female	66	Rural	15	1
P10	Female	69	Urban	13	2
P11	Female	68	Urban	12	3
P12	Male	73	Urban	8	2
P13	Male	65	Urban	6	2
P14	Male	66	Urban	6	2
P15	Male	68	Urban	8	1

Based on the interview data, we mapped the themes derived from our analysis to the dimensions of Meleis’ Transition Theory (Table 4).

**Table 4:** The connection between Meleis' transition theory, topics, and sub-topics

Meleis Transition Theory	Topics	Sub-topics
Nature of the transition	Weak perceptions and behaviors towards healthy eating	·personal habit solidification
		·negative attitudes towards nutrition management
	Lack of objective factors for independently adjusting dietary conditions	·reliance on subjective feelings
		·times of appetite change
Conditions of the transition	Personal hindrance factors	·memory impairment
		·deficiencies in self-nutrition management
	Expected external support	·assistance care support
		·ways to obtain nutritional information
Response patterns	Lack of continuous nutrition management	·interruption of professional guidance
		·avoidance of medical treatment behavior

### Weak perceptions and behaviors towards healthy eating

#### Personal habit solidification

Participants reported that post-discharge food selections were heavily influenced by personal preferences and entrenched familial dietary patterns. The ease of purchasing and choosing foods akin to those they have traditionally favored contributed to a selection bias against the adoption of healthier eating behaviors.

*I don't like oil all the time, but I don't like meat, so I eat some meatballs, and there are few fish in my house. (N1)*

*We all like to eat tofu at home, eat less fruit, and buy food repeatedly. (N6)*

*After leaving the hospital, I also had nutritious food, but I still got used to the previous food and didn't specialize in redoing it. There was still so much lard at home. (N7)*

During their hospital stays, the respondents were provided with diet education tailored to hospital settings, which catalyzed a shift from a passive to a proactive stance in managing their nutritional needs post-discharge. This transition often led to a clash between their pre-established dietary habits and the newly recommended healthy eating guidelines.

*I don't want to eat much now. I used to like fried dough sticks, but I said I should eat less. I used to like pickles, but now I'm not allowed to eat them. I'm not used to eating eggs, but I have to eat eggs. (N10)*

#### Negative attitudes toward nutrition management

Individuals residing in rural localities often subscribe to fatalistic beliefs, positing that one’s lifespan is predetermined and that adhering to a strict, modest diet could potentially extend one’s life.

*I think that everything you eat is "What has been arranged", and you always have to live longer. I believe in the popular proverb. (N1)*

Several respondents perceived a diminished appetite as an inevitable aspect of aging and remained skeptical regarding the benefits of nutritional management on their health. Their conception of nutrition management was simplistic, equating it with merely increasing food consumption, and they lacked awareness about the critical role that high-quality protein and fat play.

*Now that we are old, we can't eat anything and have no appetite. (N6)*

*There is no need to eat too well. It's easy to get sick when you gain weight, and living conditions are at an average level as long as I can live. (N9)*

*The impact of eating is too small. (N14)*

### Lack of objective factors for independently adjusting dietary conditions

#### Reliance on subjective feelings

Respondents typically gauged their physical well-being and dietary consumption based on subjective indicators like levels of comfort and sensations of gastric distension. They considered the consistent consumption of specific food categories and the timing of meals as integral components of their customary lifestyle routines, forming the foundation of their dietary management strategies. During episodes of illness, they often eschewed scientific measures to assess their health status.

*When people get old, they eat too much, their stomachs swell and they can't digest, so they can't sleep. (N15)*

*Eat less when you are uncomfortable and eat a little more when you get better. (N4).*

*Now I eat more when I am getting better, but now I still eat less (N10)*

*I can't eat when I'm not feeling well. (N12)*

#### Times of appetite change

Several participants described a transitional phase of dietary adjustment following their hospital discharge. As their health condition stabilized and their self-assessed physical state improved, there was a gradual uptick in their dietary intake.

*Generally, about one week after discharge, I feel much better, and I feel nothing (uncomfortable) (N5)*

Several respondents initially adhered to their physician’s counsel and diligently managed their diet; however, after a period of roughly ten days, a relaxation in their vigilance toward food selection, coupled with a dearth of nutritional management knowledge among family members, precipitated a decline in their dietary adherence.

*It was a little better at first, but I still didn't pay much attention to it later (after almost half a month). (N6)*

*Generally, you can eat slowly after you leave the hospital, maybe for about 10 days. Just eat with your family, and you know something you can't eat. (N7)*

### Personal hindrance factors

#### Memory impairment

The respondents experienced a progressive decline in memory attributable to aging, compromising their ability to retain information pertinent to nutrition management, and thereby adversely affecting their healthy eating practices. Aware of their diminishing cognitive capabilities, they refrained from enforcing disciplined dietary self-management. This resignation fostered attitudes of neglect and dependence among some individuals regarding their nutritional well-being.

*I can't remember a little (laughs). I have a bad memory, and I haven't taken care of it for so many years. (N4)*

*I can't remember what you just told me. (N7)*

*On the one hand, I can't remember what you told me, but I still eat what I usually eat. There is not much change, and then I forget it. (N11)*

*I only remember the general idea, but I can't remember the specific food education content. (N14)*

The respondents readily delegated their children to manage their affairs due to their acknowledged forgetfulness and longstanding reliance on family members for guidance, particularly in meal-related matters. Consequently, they neglected memory training, leading to a consistent decline in cognitive function.

*At such an old age, I have a regular life. I can't remember when my children remind me that I am too old and often need them to tell me. (N2)*

#### Deficiencies in self-nutrition management

The respondents indicated satisfaction with their current daily dietary intake, primarily evaluating adequacy in quantity and personal preference in food selection. They demonstrated a lack of comprehensive understanding regarding the specific dietary considerations essential for individuals concurrently managing coronary heart disease and frailty.

*Just eat enough and don't pay attention to what food they cook every day. (N9)*

*Now I can't eat more and more, and I don't care what I eat at every meal. (N15)*

*I don't care about this; I think the food is okay. (N12).*

When patients prepare their own meals, their focus tends to be on the convenience of the task rather than on exploring a variety of food choices and balanced combinations suitable for individuals with both coronary heart disease and frailty. Few patients take the time to educate themselves on appropriate nutrition, and some consistently adopt a perfunctory attitude towards managing their own meals three times a day.

*Sometimes I don't want to eat their cooking, so I just eat some noodles and soup myself. The food I make is convenient to make, so it's simple. (N2)*

### Expected external support

#### Assistance care support

Some of the respondents came from affluent families, where the management of their diet was overseen by either a nanny or their children. In some cases, the sole responsibility of certain nannies was to cater to the respondent’s dietary needs. The nanny or family ensured that the patient received three meals a day, and the respondent only needed to communicate their food preferences.

*What I want to eat will be bought by the nanny. Generally, what she bought is more nutritious and makes progress slowly. (N3)*

*When I leave the hospital, the nanny will cook, and I can tell the nanny and my daughter what I want to eat. (N4)*

*I have a nanny at home. The nanny lives close to us. The cooking is more delicious than that in the hospital. She helps me with meals and medicine. (N13)*

The respondents indicated a preference for professional care, placing more trust in the guidance of healthcare professionals over their own self-management post-hospital discharge. The expertise of healthcare providers is irreplaceable, as they not only offer guidance to the interviewees but also provide psychological support.

*A little more care after discharge; professional guidance is better than managing ourselves. (N11)*

### Ways to obtain nutritional information

The respondents expressed a preference for receiving dietary information in a convenient format, indicating that recipes for three meals accompanied by images were more well-received than verbal instructions from healthcare providers. While respondents were able to access brief videos on dietary considerations relevant to older adults with coronary artery disease, they faced challenges in following personalized instructions tailored.

*You can make a recipe, which looks very convenient. (N3)*

*Watching the video, it talks about what to eat and what not to eat for coronary heart disease. (N8)*

*Doctors can pay attention to it from time to time, whether they usually eat right or not and whether they have any suggestions. (N11)*

### Lack of continuous nutrition management

#### Interruption of professional guidance

After discharge from the hospital, the responsibility of overseeing the respondents’ diets transitioned from healthcare professionals to family members. The respondents hesitated to seek assistance from community health agencies when confronted with dietary concerns, leading to a void in expert guidance. The respondents noted that following their departure from the care of healthcare workers, they turned to the hospital only when they were experiencing health issues.

There is supervision in the hospital. When you leave the hospital, you can't get in touch with experts. You usually don't go to the community hospital, and you don't go to the doctor to ask what you eat. Generally, you go to the hospital when you are unwell. (N5)

Generally, if there are no major issues, I wouldn’t go to the hospital. It’s troublesome for me to go to the hospital, and it’s also troublesome for my sons.(N9)

After discharge, a little more care is needed. Professional guidance is better than self-management for us.(N11)

#### Avoidance of medical treatment behavior

After discharge, respondents did not actively seek medical assistance to address issues such as indigestion and diarrhea caused by dietary factors. They believed that discomfort arising from dietary issues is generally minor compared to the acute phase of the illness, and can be managed through personal experience or self-medication without the immediate need for medical intervention.

*You won't eat on a bad stomach at home. Even if you feel uncomfortable, just drink a little granule, and you won't ask the doctor about eating. (N4)*

*Sometimes I feel a little flustered when I eat less, so I just eat some sugar. (N12)*

## Discussion

Nature of the transition refer to the characteristics of individuals during periods of transition at different times, primarily associated with the experiences and changes during the transition process [16]. In this study, respondents revealed that their dietary choices were swayed by personal tastes and entrenched habits, which in turn evoked adverse sentiments regarding their dietary management. From the patient’s perspective, the main challenge lies in the transition from a passive role in nutritional management after discharge to an active one, causing discrepancies in patients’ nutritional management in terms of judgment, habits, and adjustments compared to the expected management process [17]. Moreover, the at-home nutritional management post-discharge cannot be monitored by a professional medical team like during hospitalization, where blood indicators are used to determine the levels of lipids and inflammatory factors affecting coronary heart disease and frailty. Consequently, providing dietary recommendations to patients at home is solely based on the subjective assessment of the disease’s recovery by the patients themselves, resulting in an increase or decrease in food intake based on their own feelings rather than utilizing appropriate nutritional indicators for self-assessment in elderly patients. First, health care professionals can use multiple indicators, such as blood, body and nutrition, as references to determine the reasonableness of dietary intake and can also use predictive models to quickly identify patients who are at risk of nutritional imbalance [18]. Second, before providing guidance regarding patients' dietary structure, health care professionals should understand patients' own long-term dietary habits, correct unfavorable intake behaviors, enrich the types of food patients can consume and optimize their dietary structure based on the original reasonable dietary structure. Finally, timely dietary management interventions during the patient's transition process require a multidisciplinary team consisting of clinicians, attending physicians, dietitians, and health managers who aim to patient recovery, improve nutritional status, and enhance organism function [19].

Conditions of the transition refer to the individual, community, and societal factors that facilitate or hinder transitions during the process of change [16]. Within this framework, this study has identified themes of personal obstacles and a desire for external support. Participants conveyed that with advancing age, they find it increasingly challenging to retain the nutritional guidance imparted by healthcare providers in their long-term memory. When respondents take on the task of nutritional management themselves, they often approach it with a casual attitude. As a result, most families either passively or actively seek nursing professionals specializing in dietary care to provide assistance to patients with their dietary needs. However, when dining at home, patients often determine their dietary intake based on their eating habits, and most family members cater to the patient's favorite foods to ensure that they eat in a timely manner, thus leading to a bias toward meat and vegetable intake [20]. Moreover, hiring a maid for the respondents requires a high level of family economic status, making it impossible for elderly patients in rural areas to enjoy this treatment and causing them to rely instead on the dietary habits and food choices of their family members. However, considering the limited literacy level, low health care awareness, and poor dietary management behaviors of patients’ families in rural areas, this situation makes it impossible to provide patients with scientific and reasonable food combining schemes [21]. Therefore, we must help patients and their families understand the benefits and harms of different types of food intake in a simple way and illustrate the nutritional composition of edible foods and a corresponding scheme using pictures and videos to reduce the burden faced by patients with regard to remembering the appropriate types of food they should consume.

In the response patterns of Meleis’ transition theory, which include process indicators and outcome indicators, methods to enhance patients’ coping with barriers to change are increased through interaction and control [16]. In the findings of this study, respondents expressed a lack of ongoing contact with professional healthcare providers after discharge. They are also unlikely to ask doctors about what to eat simply because they “don’t know what to eat.” It is evident that most respondents, when uncertain about nutritional management, do not have access to precise nutritional management strategies. More importantly, respondents fail to recognize the significance of nutritional management for long-term disease management. Therefore, various avenues for acquiring knowledge on nutrition should be provided for discharged elderly patients with coronary heart disease and frailty, such as nutrition videos on mobile phones [22] and nutrition education at community service centers [23], to help improve their nutrition awareness. In addition, caregivers, as the first contact of patients after discharge, should also pay attention to patients' behaviors that can affect healthy eating [24]. Educational interventions related to promoting nutritional management can be provided to caregiver for comparison, such as by providing patients with a clean dining environment, paying attention to the dining experience [25], identifying problems that lead to reduced diets on the part of patients with a combination of coronary heart disease and frailty due to the negative emotions associated with the fear of disease recurrence, communicating with patients in a timely manner, and reminding patients to clean their teeth or dentures with water after meals to reduce the oral cavities caused by food residue [26].

Nursing interventions are considered important interventions that influence and support the feedback of the other three elements of Meleis transition theory, providing support for improving health [16]. Through the responses of the participants, coding, categorizing, and summarizing were conducted, resulting in the formation of 10 sub-themes. Corresponding nutritional management measures can be proposed based on the sub-themes under the transition properties for future intervention nodes during the nutritional management transition period. Recommendations for nutritional knowledge on low-fat and high-quality protein intake, promoting healthy behaviors such as appetite stimulation and weight maintenance from the perspectives of patients, caregivers, and the community can be proposed based on sub-themes under the conditions of transition. Various intervention methods, such as recommending different foods in the same category and suggesting balanced meals for patients, can be provided based on sub-themes under the response patterns. Encouraging active patient participation in nutritional management during the transition period can enhance the nutritional literacy of patients with coronary heart disease and frailty.

The limitations of this study include that the interviews were conducted with elderly patients who had been discharged for more than 6 months. Elderly individuals may have memory loss and limitations in recalling their dietary management experiences. Some respondents provided vague answers regarding the nutrition management process, resulting in interviews lasting between 20 to 40 min. Without guided prompts during the interviews, researchers may not obtain sufficient information. However, it can be inferred indirectly that the respondents themselves do not place much emphasis on nutrition management. Direct relatives can help recall the types of food consumed and dietary changes, but they may not provide detailed information and may focus only on significant changes, such as dietary alterations due to physical discomfort.

## Conclusions

This study was guided by Meleis’ transition theory, with interview outlines structured based on the nature of the transition, the conditions of the transition, response patterns, to explore the experiences of nutrition management in elderly patients with coronary heart disease and frailty post-discharge. The extracted themes and sub-themes will inform the development of tailored nursing interventions for subsequent clinical applications.

The results indicate that self-awareness of health and nutrition management among elderly patients with coronary heart disease and frailty is lacking. Regardless of personal attitudes or behaviors, the respondents did not demonstrate a strong emphasis on post-discharge nutrition management. The respondents exhibited a strong dependency on caregivers, highlighting the importance of caregivers in the nutritional management outcomes of patients with coronary heart disease and frailty.

Therefore, enhancing the nutritional level and health literacy of elderly patients requires not only patient awareness of the importance of healthy eating and their actions to comply with these requirements, but also the support of family members and healthcare professionals to assist patients in health-related dietary behaviors. This includes factors such as selecting nutritious foods, objectively controlling food intake, maintaining a clean dining environment, and ensuring timely oral hygiene, all of which collectively contribute to supporting dietary management.

## Data Availability

The data results of this study can be obtained from the corresponding author as required.
